# COVID-19 pneumonia assessed at a private hospital, a field hospital, and a public-referral hospital: population analysis, chest computed tomography findings, and outcomes

**DOI:** 10.3389/fpubh.2023.1280662

**Published:** 2024-01-03

**Authors:** Patrícia Yokoo, Adham do Amaral e Castro, Eduardo Kaiser Ururahy Nunes Fonseca, Rodrigo Caruso Chate, Gustavo Borges da Silva Teles, Marcos Roberto Gomes de Queiroz, Gilberto Szarf

**Affiliations:** Hospital Israelita Albert Einstein, São Paulo, Brazil

**Keywords:** COVID-19, SARS Cov 2, epidemiology, public health, coronavirus infection

## Abstract

**Objective:**

To compare a private quaternary referral hospital, a public tertiary hospital, and a field hospital dedicated to patients with COVID-19, regarding patients’ characteristics, clinical parameters, laboratory, imaging findings, and outcomes of patients with confirmed diagnosis of COVID-19.

**Methods:**

Retrospective multicenter observational study that assessed the association of clinical, laboratory and CT data of 453 patients with COVID-19, and also their outcomes (hospital discharge or admission, intensive care unit admission, need for mechanical ventilation, and mortality caused by COVID-19).

**Results:**

The mean age of patients was 55 years (±16 years), 58.1% of them were male, and 41.9% were female. Considering stratification by the hospital of care, significant differences were observed in the dyspnea, fever, cough, hypertension, diabetes mellitus parameters, and CT score (*p* < 0.05). Significant differences were observed in ward admission rates, with a lower rate in the private hospital (40.0%), followed by the public hospital (74.1%), and a higher rate in the field hospital (89.4%). Regarding intensive care unit admission, there was a higher rate in the public hospital (25.2%), followed by the private hospital (15.5%), and a lower rate in the field hospital (9.9%). In the analysis of the discharge and death outcomes, it was found that there was a higher number of patients discharged from the private hospital (94.2%), compared to the field hospital (90.1%) and public hospital (82.3%) and a higher number of deaths in the public hospital (17.7%) compared to the private hospital and field hospital (5.8 and 0% respectively).

**Conclusion:**

The analysis of the data regarding the population treated with COVID-19 during the first wave in different levels of care in the public and private health systems in the city of São Paulo revealed statistically significant differences between the populations, reflecting distinct outcomes.

## Introduction

In February 2020, the first confirmed infection of coronavirus (COVID-19) was recorded in Brazil, and since then many advances have been achieved, ranging from preventive measures to population vaccination (1–5). The disease is now under control, and recently the WHO, after 3 years of the pandemic, declared the end of COVID-19 as a public health emergency (6).

The situation, however, was much different during the so-called first wave in the early days of the pandemic, and the national public health system and its services at various levels of complexity had to adapt to meet the needs of a new and, until then, unknown disease. Thus, to address new and evolving challenges, several large public hospitals were restructured, becoming centers dedicated to acute respiratory syndrome care. Additionally, to meet the increasing need for hospital beds, field hospitals were set up in locations such as parks and sports venues to provide initial care and hospitalization for patients with pneumonia due to the new coronavirus (7–9).

Private healthcare facilities also had to cope with a large number of suspected cases of COVID-19 acute respiratory syndrome. Faced with these conditions, the private system created specific hospitalization criteria, among these redirecting care and hospitalization flows, dedicating beds, and sometimes entire floors to these patients (10, 11).

In this context, besides RT-PCR as a confirmatory criterion for COVID-19, imaging exams, especially chest computed tomography (CT), played a major role in rapid diagnosis of airway infection and prognostic stratification, allowing effective initial patient management, both in private and public services (12–14).

Today the number of severe cases has declined substantially due to vaccination efforts; however, the data from the beginning of the pandemic remains of great value and deserve to be evaluated. Because novel viral outbreaks could occur in the future, understanding population differences, regarding their demographics and outcomes, can contribute to better planning of policies to deal with new epidemics to come (15).

This study shows the demographic characteristics of patients seen in three locations: a quaternary private hospital, a tertiary public hospital that became a reference for COVID-19 care, and a field hospital set up and dedicated exclusively to patients with COVID-19. We evaluated differences in clinical parameters, laboratory results, imaging findings, comorbidities/risk factors, and outcomes in those patients with a confirmed diagnosis of COVID-19. Our aim is to identify potential differences in patient profiles and outcomes among individuals receiving care from these facilities, distinguished by one being private and the other two being public. Insights derived from this study could help address challenges that may result in improved healthcare for our population.

## Materials and methods

### Study design

A multicenter retrospective observational study conducted in three hospital centers in the city of São Paulo, Brazil: a private quaternary reference hospital; a tertiary public hospital of reference in the care and admission of patients with new coronavirus infection; and a field hospital set up and dedicated exclusively to patients with COVID-19, under the administration of the private network under study, henceforth designated Private, Public, and Field, respectively.

Included in the study were all patients treated at one of the three participating hospitals between April 1, 2020 and April 30, 2020. Each had a RT-PCR-confirmed diagnosis of COVID-19 and had received chest CT scans.

This study was approved by the local Ethics Committees of the three institutions. An Informed Consent Form was obtained for patients seen at the tertiary hospital of the public health system, in accordance with their institutional review board’s guidelines. Patients seen at the other two sites were exempted from the ICF.

### Clinical and laboratory data

Data regarding gender, age, weight, height, body mass index, time of symptom onset, dyspnea, chest pain, oxygen saturation, abdominal symptoms, heart rate, respiratory rate, axillary temperature, systemic arterial hypertension, diabetes mellitus, chronic obstructive pulmonary disease, asthma, heart disease, smoking, and cancer were collected from medical records.

Regarding laboratory data, the CBC leukocyte series, C-reactive protein, and D-dimer were collected.

The severity of COVID-19 was assessed utilizing a modified classification derived from the one proposed by the China National Health Commission at the onset of the pandemic (16). In the scope of our research, we employed an adaptation of the described classification, with a focus on stratifying the sample between mild and severe cases. Severe cases were defined as patients meeting either the criteria of RR ≥ 30 breaths per minute or SatO2 ≤ 93%, while patients not meeting these criteria were classified as mild cases. Imaging data were not included, as the original classification relied on chest radiography. Additionally, the PaO2/FiO2 criterion was not considered in the analysis, as it was not routinely assessed at the time of patient admission.

### Imaging data and acquisition protocol

Chest CT images were acquired using 64-, 80-, or 320-detector row CT scanners (Brilliance, Philips Medical Systems, Eindhoven, Netherlands; Somaton Definition, Siemens Healthcare, Erlangen, Germany; Aquillion Prime and Aquillion ONE, Canon Medical Systems, Tochigi, Japan). All scans were acquired in the supine position at maximum inspiration, without using an intravenous contrast medium. The acquisition protocol for the CT scans had the following parameters: 1 mm slice thickness, 80–120 kVp voltage, and automatic milliamp adjustment (10–440 mA range).

Computed tomography findings were assessed by two thoracic radiologists, blinded to clinical data, at dedicated workstations with Picture Archiving and Communication System (PACS). They analyzed the images and estimated the degree of lung involvement using a semi-quantitative analysis using a scoring system (score).

The score, as described by Ooi et al., is based on the subjective visual assessment of the degree of extent of lung involvement in the form of ground-glass opacities, mosaic paving, and consolidations in six zones (three for each lung, limited by the carina and right inferior pulmonary vein planes). For each zone, a score from 0 to 4 is assigned, according to the proportion of diseased parenchyma: 0, 0%; 1, <25%; 2, 25–50%; 3, 50–75%; and 4, >75%, therefore, with a theoretical range of 0–24 (17).

Subjective evaluation of the presence of other tomographic findings was also performed; these additional presentations included emphysema, interstitial disease, bronchial wall thickening, mediastinal lymph nodes, pleural effusion, pericardial effusion, and incidental findings.

Cases were independently assessed by the two radiologists, blinded to clinical data and institution of origin, and in cases of disagreements, a final decision was reached by consensus.

### Inclusion and exclusion criteria

Inclusion criteria: participants were of both genders, 18-years-old and older, each with a diagnostic confirmation of COVID-19 by RT-PCR. They underwent at least one chest CT scan, and were seen in one of the three participating medical centers between April 1, 2020 and April 30, 2020. The first CT scan performed in the clinical setting of COVID-19 infection was analyzed.

Exclusion criteria: patients with CT scans with limited image assessment due to artifacts. Patients who did not agree to participate in the study.

### Outcomes

The outcomes evaluated were hospital discharge, hospital admission, ICU admission, need for mechanical ventilation, and death attributable to COVID-19 infection.

### Statistical analysis

Categorical variables were described by frequency and percentage tables for comparison between hospitals, and groups were compared using the likelihood ratio test. Quantitative variables were described using mean ± standard deviation or median ± interquartile range (p25; p75) and were assessed by ANOVA or *Kruskall-Wallis* tests, followed by *Bonferroni* multiple comparisons and *Dunn* multiple comparisons, respectively.

Qualitative characteristics were adjusted according to the worst outcome (need for mechanical ventilation or intensive care, or death). Absolute and relative frequencies were used, besides association with the use of the Chi-square test or likelihood ratio test, when appropriate. Quantitative characteristics were described accordingly. Summary measures were used and compared between the outcome using the *t-Student* or *Mann–Whitney test*.

IBM-SPSS software for Windows version 22.0 was used to perform the analyses and Microsoft Excel 2010 software was used to tabulate the data. Tests were considered significant with values of *p* less than 0.05.

## Results

The study’s sample was comprised of 453 patients, including 190 females and 263 males, with a mean age of 55 years (standard deviation ± 16 years). The clinical characteristics and outcomes of the patients are described (Supplementary Table 1).

As it relates to each hospital of care, significant differences were observed between the three locations. For example, dyspnea parameters were lower in the private hospital compared to the other hospitals (Private 49.0%, Field 74.5%, and Public 70.7%), while fever was higher in the field hospital (Private 64.5%, Field 82.6%, and Public 62.6%). Furthermore, lower rates of cough (Private 66.5%, Field 81.8%, and Public 82.3%), hypertension (Private 16.8%, Field 58.9%, and Public 50.3%), and diabetes mellitus (Private 9.7%, Field 35.1%, and Public 30.6%) were observed in the private hospital, with statistically significant differences.

Regarding the outcome mechanical ventilation, a statistically significant association was identified among the three hospitals (*p* = 0.003), indicating a higher incidence of mechanical ventilation in the public hospital. When applying the modified clinical severity classification, a statistically significant difference was observed among the hospitals (*p* < 0.001). In the private hospital, 14.5% of the patients were categorized as severe, whereas in the field hospital, this proportion rose to 51.7%, and in the public hospital, it reached 60.5% (Supplementary Table 1). Upon comparing only the field hospital and the public hospital, no statistically significant association was identified (*p* = 0.124).

Computed tomography characteristics of the patients included in the study are described (Supplementary Table 2). The CT score obtained in the private hospital was 6 (1,8); in the field hospital, it was 12 (9,14); and in the public hospital, it was 10 (6,12), with a statistically significant difference in the three sites (*p* < 0.05). Regarding the interval between symptoms onset and medical visit, a longer time was observed in the field hospital, with a median of 8 days (6,10), compared to the private hospital, with a median of 6 days (3,8), and the public hospital, with a median of 5 days (3,10) (*p* < 0.05).

Significant differences were observed in ward admission rates, with the lowest rate in the private hospital (40.0%), followed by the public hospital (74.1%), and the highest rate in the field hospital (89.4%). Regarding ICU admissions, the highest rate was observed in the public hospital (25.2%), followed by the private hospital (15.5%), and the lowest was seen in the field hospital (9.9%).

In the analysis of discharge and death outcomes, it was found that there was a higher number of patients discharged from the private hospital (94.2%), followed by the field hospital (90.1%) and the public hospital (82.3%), statistically significant differences. Regarding the number of deaths, the public hospital had the worst outcome (17.7%), while the private hospital and the field hospital recorded 5.8% and zero, respectively. Additionally, it is important to highlight that 9.9% of the patients seen in the field hospital were transferred to other hospitals, as their cases were more severe, and the infrastructure of that venue was limited.

Supplementary Table 3 provides a comparison of clinical parameters between the hospitals. It was found that there were differences in oxygen saturation on admission, being higher in the private hospital and lower in the public hospital (*p* < 0.001). The temperature on admission was significantly lower in the public hospital compared to the other sites (*p* < 0.05). Heart rate was higher in the public hospital compared to the field hospital (*p* < 0.001), while respiratory rate was lower in the private hospital compared to the other hospitals (*p* < 0.001). D-dimer was assessed only in the private and field hospitals, being higher in the latter (*p* < 0.001).

Regarding laboratory parameters, as shown in Supplementary Table 4, it was observed that WBC and CRP were lower in the private hospital compared to the other hospitals (*p* < 0.05).

## Discussion

This multicenter study, which examined and compared various clinical characteristics, was completed utilizing data from a sample of patients being cared for in three separate hospitals. These three facilities—a quaternary private hospital, a tertiary public hospital, and a field hospital set up and dedicated exclusively to patients with COVID-19—showed significant differences both in the population profile and outcomes.

Our data reveal that patients seen at the private hospital had a lower incidence of dyspnea and cough complaints when compared to those at the other hospitals. In addition, these patients had higher percentages of oxygen saturation, lower respiratory rates, and lower laboratory values of WBCs and PCR compared to patients seen at the other public sites. These differences are reinforced by the observation of a lower clinical severity score and a shorter time from symptom onset to hospital admission as well as a shorter duration between symptom onset and the performance of tomography in this institution. This observation is in accordance with the study by Caballer-Tarazona et al. (18), which found similar evidence of greater access to care in another private-sector clinical setting when compared to the public system.

In contrast, a longer time from symptom onset to medical visit was observed in patients seen at the field hospital, which may have contributed to the higher tomographic scores observed at this institution due to the natural progression of the disease (19), as well as the higher rate of fever in its patients compared to the other hospitals. A separate study by Amasiri et al. analyzed patients treated at a field hospital in Thailand during four waves of the pandemic and showed a predominance of asymptomatic and mildly symptomatic patients in the sample. The same outcomes were observed in other field hospitals, which is desirable due to the limited structure of those facilities, which primarily serve to deviate patients from hospitals, intentionally reserved for the most severe cases (20–22). The higher rates of individuals with fever observed in our field hospital can possibly be explained by the existence of a repressed demand of patients in the first month of operation of the field hospital. These patients accessed the health system when in more advanced stages of the disease, which also resulted in more severe image scores and more extensive lung involvement. Even taking these considerations into account, those patients seen at the field hospital had better outcomes compared to those seen at the public hospital, supporting the sense of the milder nature of their disease; this, however, is an inference, and it is not possible to measure the impact of the quality of treatment and protocols adopted there. In a similar fashion, the study by Brady et al. analyzed data from patients seen in a temporary field hospital implemented at the beginning of the pandemic in the United States. Its results revealed a lower mortality rate, including among ICU patients, compared to other reference hospitals in the same city, findings that highlight the importance of care coordination and implementation of standardized care protocols (23).

Considering two parameters of a severity score (RR ≥ 30 bpm and SatO2 ≤ 93%) (16), it can be asserted that patients treated within the public healthcare system presented a more severe clinical profile. In this context, a higher incidence of ICU admissions and an elevated mortality rate were observed. These unfavorable outcomes could be attributable to several variables. From the structural point of view, access to health services is usually easier and faster in the private system. Also, private hospitals in general have greater availability of specialized personnel (18). The public system may face challenges more often in terms of access to care, an observation supported by the study by Khalatbari-Soltani et al., which suggests a significant association between unfavorable socioeconomic factors and the risk of disease and mortality. According to this study, disadvantaged socioeconomic position played an important role in the COVID-19 pandemic, both directly and indirectly, highlighting factors related to living conditions. These impeding factors, such as lack of sanitation and adequate access to food, are also combined with and magnified by the presence of high-density households and neighborhoods, which may limit the adoption of social-distancing measures and result in greater difficulties in accessing public health services (24). The article by Moore et al. (25) also highlights that living in densely populated, low-income metropolitan areas with a lack of health insurance and limited access to health care contribute to higher rates of infection and adverse outcomes due to COVID-19.

Another aspect to be considered is related to the patient profile, as those treated in the public healthcare system present a higher rate of comorbidities, in line with the study by Pathirana et al. (26), which suggests a higher burden of multimorbidity among lower socioeconomic groups. The review article by Tynkkynen et al. (27), which compares public and private healthcare systems in Europe, concludes that in terms of quality, there is no significant data demonstrating superiority of either system. However, it has been shown that the patient profile treated in public hospitals consists of older patients, with more comorbidities, less favorable economic conditions, riskier lifestyles, and more complications when compared to patients treated in private hospitals.

The considerations and limitations that should be highlighted for the interpretation of the described results are as follows: that the sample was collected retrospectively at the beginning of the pandemic, before the beginning of vaccination against COVID-19; and indirect statistical inferences were made, where neither specific care protocols nor the cause of death was evaluated.

Nevertheless, the results of this study reflect the situation in Brazil at the beginning of the pandemic, where different health systems were operating, and are relevant for understanding their impact on the population. That comprehension can contribute to improving protocols and the management of future events, guiding actions to better assist the varied populations utilizing different health care facilities.

## Conclusion

The analysis of the data revealed significant disparities and equally distinct outcomes between three populations treated for COVID-19 in the city of São Paulo during the first wave of the pandemic. Different levels of care were observed at the three facilities. Opportunities for exploration, particularly in enhancing outcomes within the Brazilian public health system during future infectious disease outbreaks, depend on facilitating patient access to the healthcare system for prompt intervention, improving the management of chronic diseases, and reevaluating patient-care protocols alongside resource availability.

## Data availability statement

The original contributions presented in the study are included in the article/Supplementary material, further inquiries can be directed to the corresponding author.

## Ethics statement

The studies involving humans were approved by Ethics Committees of Hospital Israelita Albert Einstein and Municipal Health Department of São Paulo. The studies were conducted in accordance with the local legislation and institutional requirements. Written informed consent for participation was not required from the participants or the participants’ legal guardians/next of kin in accordance with the national legislation and institutional requirements.

## Author contributions

PY: Project administration, Writing – original draft. AC: Supervision, Writing – review & editing. EF: Writing – review & editing. RC: Supervision, Writing – review & editing. GT: Conceptualization, Investigation, Supervision, Writing – review & editing. MQ: Supervision, Writing – review & editing. GS: Conceptualization, Project administration, Supervision, Writing – review & editing.
