# Supplementary material for: COVID-19 pneumonia assessed at a private hospital, a field hospital, and a public-referral hospital: population analysis, chest computed tomography findings, and outcomes
Source: Front Public Health. 2024 Jan 3;11:1280662. doi: 10.3389/fpubh.2023.1280662 (PMC10793654; doi:10.3389/fpubh.2023.1280662)
Supplement: Supplementary file 1 [file Table_1.DOCX]

Supplementary Material

**Table 1*.*** Clinical characteristics and outcomes of the patients included in the study

| **Variable** | **Hospital** | | | **p-value** |
| --- | --- | --- | --- | --- |
|  | Private | Field | Public |  |
| Age (years) | 54.1 ± 17.2 | 56.3 ± 14.5 | 54.2 ± 15.9 | 0.419 |
| Gender (male) | 95 (61.3) | 84 (55.6) | 84 (57.1) | 0.582 |
| BMI (kg/m^2^) | 27.3 ± 4.3 | 30.6 ± 5.1 | 20.4 ± 0 | **0.028** |
| Dyspnea | 76 (49.0) | 111 (74.5) | 104 (70.7) | **<0.001** |
| Abdominal symptoms | 31 (20.0) | 30 (20.1) | 31 (21.1) | 0.968 |
| Fever | 100 (64.5) | 123 (82.6) | 92 (62.6) | **<0.001** |
| Cough | 103 (66.5) | 121 (81.8) | 121 (82.3) | **0.001** |
| Other symptoms | 95 (61.3) | 105 (69.5) | 85 (57.8) | 0.098 |
| Hypertension | 26 (16.8) | 89 (58.9) | 74 (50.3) | **<0.001** |
| Diabetes | 15 (9.7) | 53 (35.1) | 45 (30.6) | **<0.001** |
| COPD | 2 (1.3) | 6 (4.0) | 3 (2.0) | 0.302 |
| Asthma | 9 (5.8) | 6 (4.0) | 4 (2.7) | 0.392 |
| Heart disease | 21 (13.5) | 13 (8.6) | 10 (6.8) | 0.404 |
| Smoker |  |  |  | 0.121 |
| No | 137 (88.4) | 135 (89.4) | 119 (81.0) |  |
| Yes | 3 (1.9) | 4 (2.6) | 2 (1.4) |  |
| Ex-smoker | 15 (9.7) | 12 (7.9) | 26 (17.7) |  |
| Neoplasia | 10 (6.5) | 3 (2.0) | 4 (2.7) | 0.096 |
| Obesity | 36 (23.8) | 30 (19.9) | 12 (8.2) | **0.001** |
| Other comorbidities | 41 (26.5) | 35 (23.2) | 26 (17.7) | 0.185 |
| ICU | 24 (15.5) | 15 (9.9) | 37 (25.2) | **0.002** |
| Ward | 62 (40.0) | 135 (89.4) | 109 (74.1) | **<0.001** |
| Outcome |  |  |  | **<0.001** |
| Discharge | 146 (94.2) | 136 (90.1) | 121 (82.3) |  |
| Death | 9 (5.8) | 0 (0.0) | 26 (17.7) |  |
| Transfer | 0 (0.0) | 15 (9.9) | 0 (0.0) |  |
| Sp02 (%) | 95.6 ± 3 | 93.8 ± 3.2 | 91.2 ± 5.6 | **<0.001** |
| Temperature (Celsius) | 36.8 ± 0.9 | 36.9 ± 1 | 36.5 ± 0.7 | **<0.001** |
| Heart rate (beats/min) | 90.7 ± 15.2 | 87.8 ± 14.9 | 94.9 ± 15.8 | **0.001** |
| Respiratory rate (breaths/min) | 18.5 ± 3.7 | 21.9 ± 4.3 | 21.3 ± 4.8 | **<0.001** |
| Leukocyte series (µL) | 5420 (4097.5; 7685) | 6900 (5280; 8980) | 7510 (5705; 9135) | **<0.001** |
| C-reactive protein (mg/dL) | 28.8 (7.3; 67.2) | 75 (37; 147) | 90.8 (36; 172.2) | **<0.001** |
| D-dimer (ng/mL) | 457 (304.5; 780) | 675 (407; 1342) |  | **<0.001** |
| MV | 17 (11.0) | 14 (9.3) | 32 (21.8) | **0.003** |
| Risk |  |  |  | **<0.001** |
| Mild | 130 (85.5) | 72 (48.3) | 58 (39.5) |  |
| Severe | 22 (14.5) | 77 (51.7) | 89 (60.5) |  |
| Data are expressed as n (%), mean ± standard deviation or median (p25; p75) | | | |  |
