# Supplementary material for: COVID-19 pneumonia assessed at a private hospital, a field hospital, and a public-referral hospital: population analysis, chest computed tomography findings, and outcomes
Source: Front Public Health. 2024 Jan 3;11:1280662. doi: 10.3389/fpubh.2023.1280662 (PMC10793654; doi:10.3389/fpubh.2023.1280662)
Supplement: Supplementary file 2 [file Table_2.DOCX]

Supplementary Material

**Table 2 -** Tomographic characteristics of the patients included in the study

| **Variable** | **Hospital** | | | **p-value** |
| --- | --- | --- | --- | --- |
|  | Private | Field | Public |  |
| Distribution |  |  |  | **<0.001** |
| Absent | 34 (21.9) | 6 (4) | 4 (2.7) |  |
| Undistributed | 40 (25.8) | 57 (37.7) | 87 (59.2) |  |
| Central | 7 (4.5) | 2 (1.3) | 4 (2.7) |  |
| Peripheral | 58 (37.4) | 33 (21.9) | 33 (22.4) |  |
| Diffuse | 16 (10.3) | 53 (35.1) | 19 (12.9) |  |
| Location |  |  |  | **<0.001** |
| Posterior | 32 (26.4) | 12 (8.3) | 27 (18.9) |  |
| No predominant | 89 (73.6) | 131 (90.3) | 116 (81.1) |  |
| Anterior | 0 (0) | 2 (1.4) | 0 (0) |  |
| Predominant finding |  |  |  | **<0.001** |
| Absent | 34 (22.1) | 6 (4) | 4 (2.7) |  |
| Ground-glass opacity | 114 (74) | 128 (84.8) | 131 (89.1) |  |
| Consolidation | 6 (3.9) | 17 (11.3) | 12 (8.2) |  |
| Emphysema | 7 (4.5) | 17 (11.3) | 19 (12.9) | **0.030** |
| Interstitial disease | 2 (1.3) | 3 (2) | 1 (0.7) | 0.604 |
| Mediastinal lymph nodes | 9 (5.8) | 54 (35.8) | 34 (23.1) | **<0.001** |
| Pleural effusion | 16 (10.3) | 41 (27.2) | 24 (16.3) | **0.001** |
| Pericardium | 4 (2.6) | 11 (7.3) | 8 (5.4) | 0.168 |
| Structured classification |  |  |  | **<0.001** |
| negative | 36 (23.2) | 6 (4) | 4 (2.7) |  |
| atypical | 1 (0.6) | 1 (0.7) | 9 (6.1) |  |
| indeterminate | 31 (20) | 10 (6.6) | 16 (10.9) |  |
| typical | 87 (56.1) | 134 (88.7) | 118 (80.3) |  |
| Neoplasia | 0 (0) | 1 (0.7) | 2 (1.4) | 0.235 |
| Score | 6 (1; 8) | 12 (9; 14) | 10 (6; 12) | **<0.001** |
| Data are expressed as n (%) or median (p25; p75) | | | | |
