# Supplementary material for: COVID-19 pneumonia assessed at a private hospital, a field hospital, and a public-referral hospital: population analysis, chest computed tomography findings, and outcomes
Source: Front Public Health. 2024 Jan 3;11:1280662. doi: 10.3389/fpubh.2023.1280662 (PMC10793654; doi:10.3389/fpubh.2023.1280662)
Supplement: Supplementary file 3 [file Table_3.DOCX]

Supplementary Material

**Table 3 -** Comparison of clinical parameters between the hospitals

| **Variable** | **Comparison** | | **Mean difference** | **Standard error** | **p-value** | **CI (95%)** | |
| --- | --- | --- | --- | --- | --- | --- | --- |
|  |  |  |  |  |  | Lower | Upper |
| SpO2 (%) | Private - | Field | 1.81 | 0.48 | **<0.001** | 0.67 | 2.96 |
|  | Private - | Public | 4.38 | 0.48 | **<0.001** | 3.24 | 5.53 |
|  | Field - | Public | 2.57 | 0.48 | **<0.001** | 1.41 | 3.73 |
| Temperature  (Celsius) | Private - | Field | -0.16 | 0.11 | 0.430 | -0.41 | 0.10 |
|  | Private - | Public | 0.28 | 0.10 | **0.017** | 0.04 | 0.53 |
|  | Field - | Public | 0.44 | 0.11 | **<0.001** | 0.18 | 0.69 |
| Heart rate  (beats/min) | Private - | Field | 2.89 | 1.87 | 0.372 | -1.61 | 7.38 |
|  | Private - | Public | -4.24 | 1.77 | 0.051 | -8.49 | 0.01 |
|  | Field - | Public | -7.13 | 1.89 | **0.001** | -11.67 | -2.58 |
| Respiratory rate (breaths/min) | Private - | Field | -3.38 | 0.51 | **<0.001** | -4.59 | -2.16 |
|  | Private - | Public | -2.81 | 0.49 | **<0.001** | -3.99 | -1.63 |
|  | Field - | Public | 0.57 | 0.51 | 0.810 | -0.66 | 1.79 |
