# Supplementary material for: COVID-19 pneumonia assessed at a private hospital, a field hospital, and a public-referral hospital: population analysis, chest computed tomography findings, and outcomes
Source: Front Public Health. 2024 Jan 3;11:1280662. doi: 10.3389/fpubh.2023.1280662 (PMC10793654; doi:10.3389/fpubh.2023.1280662)
Supplement: Supplementary file 4 [file Table_4.DOCX]

Supplementary Material

**Table 4 –** Comparison of laboratory parameters between the hospitals

| **Variable** | **Comparison** | | **p-value** |
| --- | --- | --- | --- |
|  |  |  |  |
| Leukocyte (µL) | Private - | Field | **0.002** |
|  | Private - | Public | **<0.001** |
|  | Field - | Public | 0.448 |
| C-reactive protein  (mg/dL) | Private - | Field | **<0.001** |
|  | Private - | Public | **<0.001** |
|  | Field - | Public | 0.325 |
| Score | Private - | Field | **<0.001** |
|  | Private - | Public | **<0.001** |
|  | Field - | Public | **0.002** |
